# Prescription opioid dispensing in New South Wales, Australia: spatial and temporal variation

**DOI:** 10.1186/s40360-018-0219-0

**Published:** 2018-06-18

**Authors:** M Mofizul Islam, Ian S. McRae, Soumya Mazumdar, Paul Simpson, Dennis Wollersheim, Kaniz Fatema, Tony Butler

**Affiliations:** 1 Department of Public Health, La Trobe University, Melbourne, Victoria, Australia; 2 Research School of Population Health, ANU College of Health and Medicine, Australian National University, Canberra, Australia; 3 Healthy People and Place Unit, Population Health, Liverpool Hospital, South West Sydney Local Health District, New South Wales Health, Sydney, New South Wales, Australia; 4 The Kirby Institute, University of New South Wales, Sydney, Australia; 5 Centre for Health Equity Training, Research & Evaluation (CHETRE), Sydney, New South Wales, Australia; 6 UNSW Sydney Research Centre for Primary Health Care & Equity, Sydney, New South Wales, Australia; 7 Population Health, South Western Sydney Local Health District, NSW Health, Sydney, New South Wales, Australia; 8 Ingham Institute, Liverpool, New South Wales, Australia

**Keywords:** Opioid, Dispensing, Prescription opioid, Pain medicine, New South Wales, Australia

## Abstract

**Background:**

Patterns of opioid dispensing often exhibit substantial temporal and geographical variability, which has implications for public health policy decisions and interventions. The study examined recent trends in prescription opioid dispensing and identified high dispensing areas and factors associated with the doses dispensed.

**Methods:**

Three years (1 January 2013–31 December 2015) of dispensing data of prescription opioids in local government areas (LGAs) for New South Wales (NSW), Australia’s most populous state, were analyzed. The proportion of individuals who were dispensed opioids was computed for four age-groups. A Chi-square test was used to examine trends over time in proportions of the population who were dispensed opioids in four age-groups. The number of prescriptions over time and quantities in daily defined dose/1000 people/day (denoted DDD) were also examined. LGAs with relatively high levels of dispensing were identified and mapped. A multivariate regression model was used to identify factors associated with DDD.

**Results:**

Overall, codeine, oxycodone and tramadol were the main opioids in terms of DDD, number of prescriptions and number of individuals who were dispensed these medications. Quantity (in DDD), and population dispensed to were consistently higher for women than men over time. Proportions of individuals who were dispensed opioids increased significantly over time in all four age-groups. In the multivariate model, age, urbanization, sex and socio-economic indexes for areas were significantly associated with doses dispensed among opioid users. All areas with very high dispensing were outside major metropolitan areas.

**Conclusions:**

Given that over-use of opioids is a major public health problem and that long-term use has substantial side effects including dependence, it is important to understand spatial patterns of opioid prescribing to enable targeted interventions. Nationwide implementation of real-time drug-monitoring programs and access to monitoring databases from both doctor and pharmacy point-of-care sources may potentially reduce excessive and undue use of opioid.

## Background

Opioids are primarily prescribed for pain relief, and occasionally for reducing coughing and relieving diarrhea. However, these drugs have substantial addictive properties as they produce feelings of euphoria, tranquility, and sedation that may lead to dependence [1, 2]. Rates of nonmedical use, misuse, and fatal poisoning from prescription opioids have increased alongside the medical use of these drugs over recent years [3]. Between 2011 and 2015 in Australia, 3601 people died from opioid-related overdose – a nearly twofold increase from 2001 to 2005 – with Fentanyl being the most common opioid implicated in these deaths [4, 5]. Although the relationship between opioid prescriptions and the risk of overdose and death is widely appreciated [6, 7], opioid prescribing practices, misuse and diversion exhibit substantial geographical variability which may have implications for public health policy decisions and interventions [8–10]. For instance, data show that the patterns of diversion, misuse and/or unsanctioned use of prescription opioid differ across Australian states and territories [11]. This spatial variation in usage is likely to be influenced by factors such as socio-economic status, age and sex of residents. Studying the relationships between patterns of opioid prescribing, quantity dispensed and population characteristics of those who use opioids at relatively small geographies could be useful for policy development. This information can facilitate understanding of where inappropriate dispensing, prescribing, and prescription opioid-seeking behaviors may occur and offer valuable information for implementation of local-level interventions.

Geographical variation in the prescription of opioids has been widely examined in the United States of America (USA) and found to be helpful in tailoring policy and interventions [9, 12–14]. Federal agencies, such as the Centers for Disease Control and Prevention, and the Drug Enforcement Agency, use a geographical information system (GIS) platform to coordinate efforts to address problematic drug use and supply across the USA. This platform is an infrastructure for the free flow of geographic data between local law enforcement, health agencies, and communities [15]. However, in other countries there have been few geographic studies that explore prescribing patterns at local level. This has been largely attributed to privacy concerns that make it difficult to obtain data for small areas [16]. In Australia, research conducted on prescription opioid utilization mostly relies on national or state level data [17, 18], with little or limited focus on small geographical areas [19]. Identifying locations with relatively high levels of prescription use (defined by unexpectedly high levels of dispensing) on smaller spatial scales is an important first step in describing the use/misuse of prescribed opioids [16]. This study examines (i) trends in prescription opioid dispensing in New South Wales (Australia’s most populous state), (ii) variation in the distribution of dispensing in local government areas (LGAs) to identify the areas with relatively high level of dispensing, and (iii) identifies factors associated with the quantity dispensed per person.

## Method

### Prescription opioid dispensing in Australia

Under Australia’s universal healthcare scheme – Medicare – around 80% of all prescription medicines dispensed are subsidized by the Pharmaceutical Benefits Scheme (PBS) [20]. In the PBS the maximum price of a single prescription (i.e. the patient contribution) in 2017 is AU$38.80 for general patients, and $6.30 for those holding government concession cards. There is also a safety net provision on PBS medications such that when a certain threshold (defined as the total patient copayments for a family) is reached in a calendar year, general patients are entitled to PBS medications at the concession price and concessional patients are entitled to PBS medications at no cost – for the remainder of the year. There is also a parallel, albeit small, Repatriation Pharmaceutical Benefits Scheme (RPBS) for military veterans, which is managed and funded by the Department of Veterans’ Affairs, that entitles this group to the same copayments as concessional patients. If a medicine is not listed in the PBS schedule (or RPBS), the patient pays the full price as a private prescription and this cannot count towards the safety net threshold [20] and is not included in this data set. A number of PBS items are priced below the maximum co-payment for general patients, and thus patients pay the full cost for those items. This payment contributes to the safety net threshold and these prescriptions are included in this data set.

### Analysis

Prescription opioid data on dispensing episodes under the PBS/RPBS were provided by the statistics branch of the Australian Department of Human Services. This study covered data for NSW and the Australian Capital Territory (which was treated as an LGA for the purposes of this study and was included in any reference to NSW data hereafter). The dataset contained information about users’ sex and age-groups (0–19 years, 20–44 years, 45-64 years, and 65+ years); generic name, form and strength of opioids; the quantity dispensed; number of prescriptions dispensed; number of persons dispensed; LGA in which the script was dispensed; and scheme (i.e. PBS, RPBS and Under co-payment). The quantity of each drug dispensed was estimated in daily defined dose/1000 people/day (indicated as DDD). The DDD unit was established by the World Health Organization Collaborating Centre for Drug Statistics Methodology [21] and corresponds to an estimated mean daily dose of the drug when used for its main indication in adults. It allows for simple comparisons of medicine use across countries, and across different formulations of the medicine. The DDD is defined by the formula $\frac{N\times M\times Q}{DDD\  factor\times P\times D}$ × 1000 [22];

where *N* is the number of prescriptions dispensed for a specific drug in a year, *M* is the mass of each dose (e.g. milligrams or grams, expressed in the same unit as DDD factor), *Q* is the average dispensed quantity per prescription, *P* is the population for the year of data collection, *D* is the number of days in the year [22].

Population data for individual LGAs and Socio-Economic Indexes for Areas (SEIFA) which ranks areas according to relative socio-economic disadvantage were obtained from the Australian Bureau of Statistics. SEIFA data are derived from the information collected in five-yearly national census. Higher scores on the Index of Relative Socio-economic Disadvantage indicate a lower level of disadvantage and lower scores mean a higher level of disadvantage. The proportion of individuals who were dispensed opioids was computed for four age-groups and for individual years from 2013 and 2015. A Chi-square test for trend of proportions was used to determine significant differences over time. LGA urbanization level was determined by the Australian Classification of Local Government, as categorized in 2013 [23].

The differences between “average DDD-quantities dispensed for overall NSW” and “age and sex adjusted DDD-quantities dispensed for individual LGAs” were estimated to identify LGAs with relatively high dispensing rates. LGAs were identified as “normal” if dispensing was less than or equal to the state average; “high” if dispensing was between state average and the third quartile; and “very high” for anything more than that. LGAs of these three categories were then mapped using R software. Trends in dispensing quantities (DDDs) over time were categorized as “increasing”, “decreasing” and “mixed”.

To examine possible factors associated with the quantity of doses dispensed, a multivariable regression model was developed. As the variable “DDD” was positively skewed we used a logarithmic transformation. To make the coefficients (β) of the regressions easily interpretable they were exponentially transformed and reported as value ‘B’. The interpretation of B is that a one unit increase (e.g. from zero to one) of the independent variable would result in a (B-1)*100 percentage change in the quantity of prescription opioids dispensed per person. The index of relative socio-economic disadvantage scores was divided into quartiles for modelling purposes, as a linear relationship does not exist.

STATA SE 14 (StataCorp LP) [24], Microsoft Office Excel 2013 [25] and R [26] were used for analyses. The study received approval from La Trobe University’s Ethics Committee (reference number: S17–218). Written informed consent was not required as these are de-identified secondary data.

## Results

**Fig. 1:**
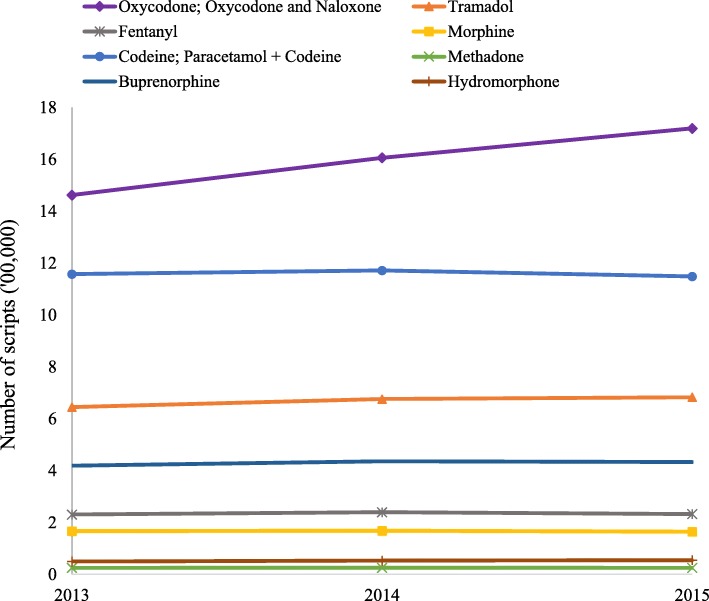
Annual trends in number of scripts between 2013 and 2015

Opioid dispensing increased in NSW across the study period (1 January 2013–31 December 2015) in terms of number of prescriptions, and number of individuals to whom opioids were dispensed but remained similar in terms of DDD (Fig. 1, Tables 1 and 2). During the three years more than 17 million opioid prescriptions were filled. Codeine and its derivatives dominate the quantity (in DDDs) of individual items dispensed followed by buprenorphine and oxycodone (and derivatives) (Table 1). However, in terms of the number of prescriptions, oxycodone (and derivatives) was the most commonly prescribed with an increase between 2013 and 2015 (Fig. 1). The rate of increase in the number of individuals dispensed prescription opioids was highest for oxycodone, although more individuals were dispensed codeine (and derivatives) (Table 2). Overall, in all three measures, codeine, oxycodone and tramadol were the main opioids dispensed in NSW.

**Table 1:** Quantity of major prescription opioids dispensed in PBS/RPBS and under-copayment scheme (in DDD)

Generic drugs	2013	2014	2015
Tramadol	2.53	2.57	2.55
Codeine and derivatives^a^	6.12	6.10	5.86
Oxycodone and derivatives^b^	3.64	3.62	3.56
Fentanyl	1.32	1.36	1.32
Morphine (& derivatives)	0.90	0.86	0.79
Buprenorphine	0.61	0.62	0.61
Hydromorphone	0.36	0.38	0.38
Methadone	0.32	0.31	0.30
Tapentadol^c^	–	0.05	0.26

^a^Codeine and paracetamol combination; ^b^Oxycodone and naloxone combination; ^c^Tapentadol was listed in Pharmaceutical Benefits Scheme from 2014

**Table 2:** Annual trends in terms of number of individuals who were dispensed opioids (in 100,000)

Year	Oxycodone and derivatives^b^	Tramadol	Fentanyl	Morphine (and derivatives)	Codeine and derivatives^a^	Methadone	Buprenorphine	Hydromorphone
2013	4.42	1.86	0.49	0.41	5.93	0.03	0.76	0.12
2014	4.95	1.94	0.49	0.42	6.02	0.03	0.76	0.12
2015	5.28	1.94	0.46	0.42	5.93	0.03	0.73	0.13

^a^Codeine and paracetamol combination; ^b^Oxycodone and naloxone combination

The quantity of opioids dispensed in terms of DDD was consistently higher for women than men across the years (Table 3). Also, more women were prescribed opioids than men. On average in each year of 2013–2015 around 808, 017 women received prescriptions compared to 659, 880 men. This difference was also reflected in the number of prescriptions per user, which was 37% higher for women than men.

**Table 3:** Opioids dispensed (in DDDs) in PBS/RPBS and under-copayment scheme across four years stratified by SEIFA and Gender

Year	Quantity dispensed in terms of DDD					
	SEIFA				Gender	
	Low	Moderate	High	Very high	Male	Female
2013	19.21	20.05	18.79	9.99	14.56	17.03
2014	19.51	20.21	18.85	10.00	14.65	17.13
2015	19.02	20.05	18.74	9.75	14.32	16.94

**Table 4:** Dispensing of prescription opioids across the years in four age-groups

	Variable	Age-group			
Year		0–19	20–44	45–64	65+
2013	Population (‘00,000)	19.58	27.38	19.26	11.68
	Person used prescription opioids (‘00,000)	0.35	3.87	4.54	5.25
	Proportion used prescription opioids (%)	1.78	14.13	23.57	44.95
	Amount of prescription opioids used (DDD)	0.31	8.02	26.23	42.83
2014	Population (‘00,000)	19.77	27.73	19.46	12.06
	Person used prescription opioids (‘00,000)	0.37	4.01	4.77	5.58
	Proportion used prescription opioids (%)	1.78	14.46	24.51	46.26
	Amount of prescription opioids used (DDD)	0.33	7.89	26.30	43.05
2015	Population (‘00,000)	19.95	28.09	19.67	12.43
	Person used prescription opioids (‘00,000)	0.37	4.17	4.90	5.75
	Proportion used prescription opioids (%)	1.85	14.84	24.91	46.26
	Amount of prescription opioids used (DDD)	0.31	7.51	25.88	42.45

There was a clear age trend in dispensing with consistently highest usage for people aged 65 years or older (Table 4). Almost half (45%) of the total NSW population aged 65 years or older were dispensed opioid. Among the 45–64 age-group around a quarter used opioids across the years. Proportions of individuals who were dispensed opioids increased significantly across 2013 to 2015 for all four age-groups and the rate of increment is similar.

Small differences in DDD quantities across LGAs of low to high SEIFA were observed, although the quantities decreased for very high SEIFA score LGAs (least disadvantaged) (Table 3). Multivariate modelling showed a significant pattern of more opioid use in the least advantaged areas and less use in the most advantaged (Table 5).

**Table 5:** Multivariate model reflecting factors associated with the DDD dispensed

Variables	B^a^	*p*	95% Confidence Interval
Sex			
Men	1.00	–	–
Women	1.13	< 0.01	1.11–1.15
Age-group (years)			
0–19	1.00	–	–
20–44	4.23	< 0.01	4.06–4.40
45–64	7.98	< 0.01	7.68–8.30
65+	11.39	< 0.01	10.96–11.84
Year			
2013	1.00	–	–
2014	0.97	< 0.01	0.95–0.99
2015	1.02	0.04	1.00–1.04
SEIFA index of disadvantage			
Low	1.00	–	–
Moderate	0.88	< 0.01	0.86–0.90
High	0.85	< 0.01	0.83–0.87
Very high	0.63	< 0.01	0.61–0.64
Urbanization			
Urban	1.00	–	–
Rural	4.08	< 0.01	4.00–4.16
Constant	1.80	< 0.01	1.73–1.88

^a^Exponentiated values of the coefficients estimated in the linear model of the logarithm of quantity of opioids dispensed

Dispensing (in DDDs) was consistently higher in rural areas than in urban areas across the years with a 300% difference in the doses of prescription opioid dispensed between the people in urban areas and the people in rural areas (B = 4.08, CI: 4.00–4.16) (Table 5).

Multivariate regression identified that women were more likely to be dispensed higher quantities of prescription opioids than men. Coefficients increased with age and peaked for the 65+ years age-group (Table 5).

**Fig. 2:**
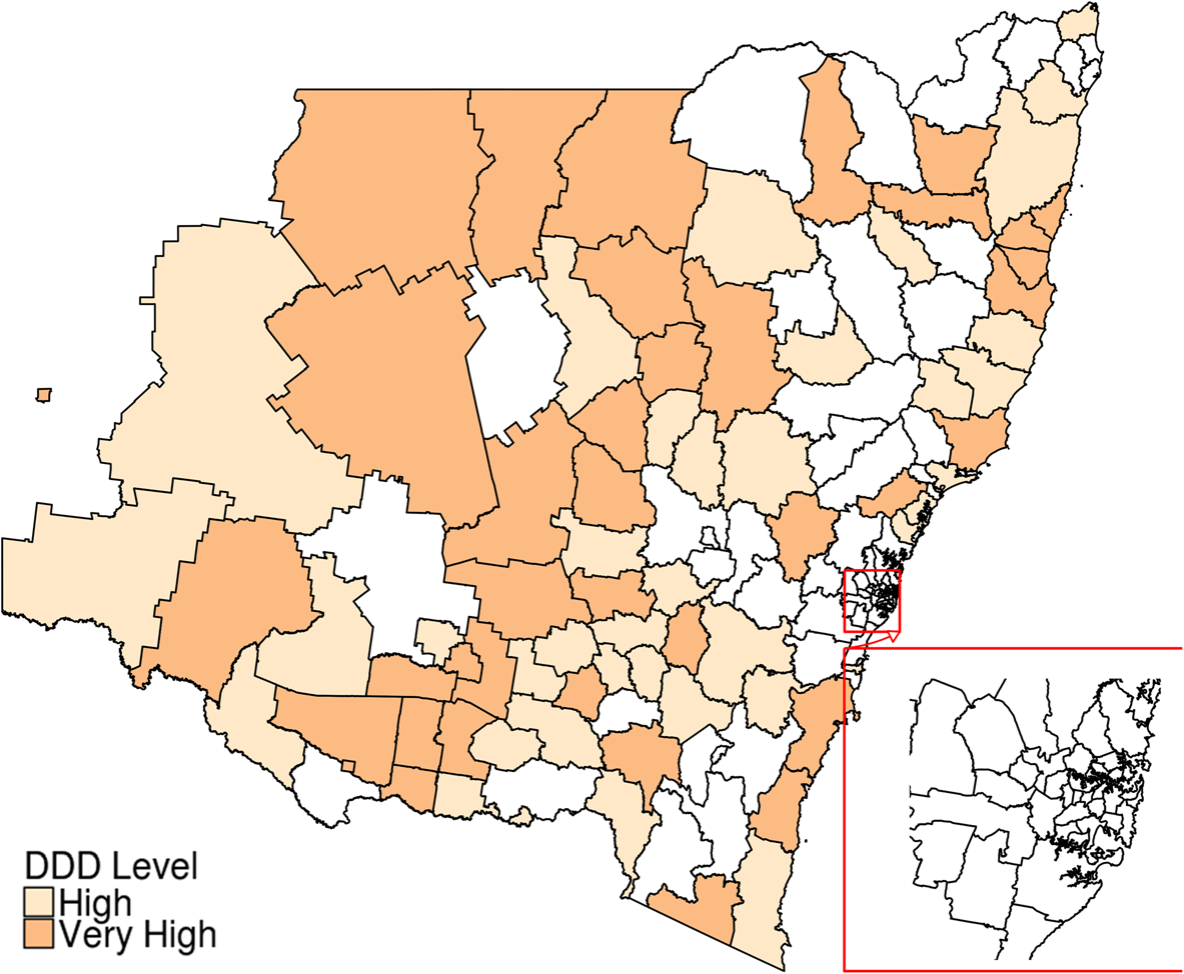
LGAs with relatively high dispensing of prescription opioid in 2015. (This map has been developed using bioregional assessment source dataset, available under a Creative Commons Attribution license. Australian Bureau of Statistics (2011) Local Government Areas of Australia. Bioregional Assessment Source Dataset)

**Fig. 3:**
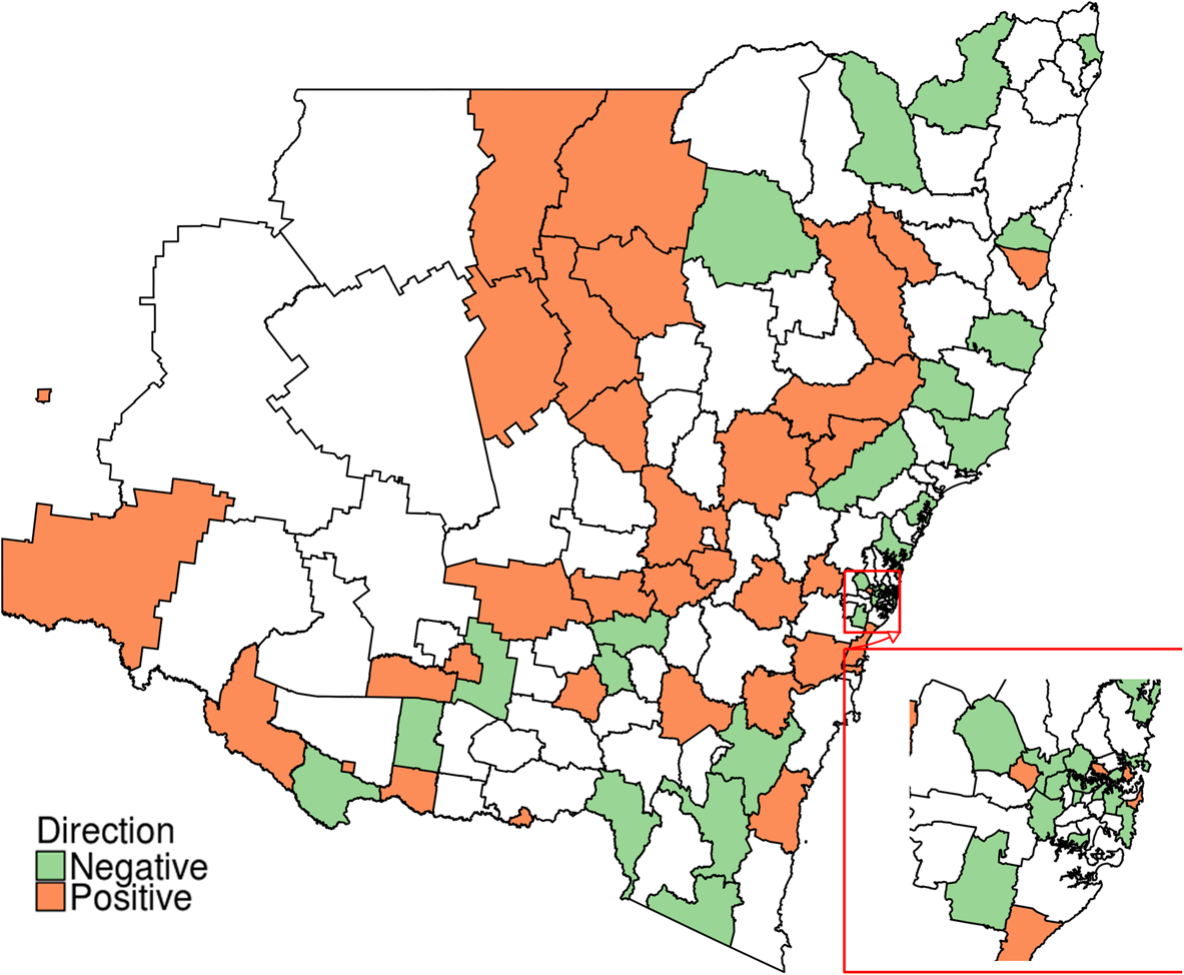
LGAs with increasing and decreasing trends in quantities (DDD) in year 2013–2015. (This map has been developed using bioregional assessment source dataset, available under a Creative Commons Attribution license. Australian Bureau of Statistics (2011) Local Government Areas of Australia. Bioregional Assessment Source Dataset)

The average quantity of dispensing in DDDs, adjusted for age and sex was mostly similar between 2013 and 2015. Taking all 154 LGAs together, it was estimated that in 2015 on an average 21.17 DDDs were dispensed per LGA (range 5.01 to 45.58, SD = ±9.43). The opioid dispensing (in DDDs) for individual LGAs were “very high” relative to the state average for 39 LGAs, “high” for 38 LGAs and “normal” for the rest of the state’s LGAs (Fig. 2), using the definitions outlined in the Methods section. Most areas with “high” or “very high” dispensing were outside of the major metropolitan areas. Quantities (in DDD) dispensed between 2013 and 2015 had mixed trends in different LGAs, increasing for 37 and decreasing for 35 LGAs (Fig. 3).

## Discussion

The findings of this study suggest that considerable variation exists in the dispensing of prescription opioids across LGAs in NSW. Overall, there was a slightly increasing trend in the number of scripts written for, and persons prescribed, opioids between 2013 and 2015. However, quantities, expressed in DDDs, were similar during this period. This is likely because the slight increase in prescriptions was mostly for opioids with relatively low DDDs such as hydromorphone and combination of oxycodone with naloxone. Women were more likely to be dispensed opioids than men. The proportion of the population dispensed an opioid increased across all age-groups over the study period. Prescriptions for oxycodone plus naloxone (in combination) rose faster than for any other opioid formulation between 2013 and 2015.

Multiple reasons explain the overall increasing trend in the proportion of citizens using prescription opioid in NSW such as an increase in the prevalence and severity of conditions causing chronic pain due to the ageing population [27]. Awareness of pain relief has increased substantially over recent years. Globally, medical, ethical and legal attention and initiatives have emerged suggesting that pain management is a fundamental human right. Such a view acknowledges that failure to treat pain is poor medicine, unethical practice, and contravenes a person’s human rights [28]. The cultural precept that “all suffering should be avoided” also encourages patients to seek opioids and the physicians to prescribe them to reduce pain [29]. In most western countries, general practitioners follow the so-called customer service norm rather than a patient care driven practice of medicine. Patients should not be treated only as customers but also as patients and clients. These two views are usually but not always synergetic. An excessive attention to customer satisfaction may encourage the over prescription of opioid-based pain relief [29]. Writing a prescription for opioids is fast and easy, whereas developing a comprehensive pain management plan is complex and seldom financially rewarded in the primary health care setting. Moreover, new products including slow-release formulations have been introduced with aggressive marketing by pharmaceutical companies [30]. Illicit substance users’ increasing reliance on prescription opioids may also be implicated in the increasing trend in opioid dispensing [31, 32].

The sharp increase in the number of prescriptions for oxycodone is likely to be due to a mixture of increased prescribing for serious pain relief and increased prescribing for people seeking the medication for non-medical purposes (e.g., recreational use). These changes can be attributed to factors such as formulations with various routes of administration; increased euphoric effects (relative to some other opioid items); illicit substance users’ increasing reliance on prescription opioids driven by the relatively low price and easy access [33], and/or a shortage of and price increases in heroin or other more traditional illicit drugs. [34]. Aggressive marketing by pharmaceutical companies also has an impact [30]. Having both immediate release formulations, which work quickly to ease pain, and slow-release formulations, which release oxycodone over several hours to provide a more even pain control could be another reason. Part of this increased dispensing could be attributed to the fact that oxycodone is sometimes used in combination with naloxone, which helps to reduce some of the side-effects of oxycodone such as constipation [35] and makes this formulation more suitable than other generic items.

Our findings suggest that women are more likely than men to be prescribed opioids which is consistent with previous studies [7, 36, 37] and likely due to a greater propensity among women to seek treatment, report symptoms and live longer than men. Although recreational use of prescription opioids by men is much higher than women [38] according to the Centers for Disease Control and Prevention, the likelihood of experiencing pain is higher among women, and so is utilization of pain medications [39]. Also, women use these medications for longer duration and in higher doses than men. A US study found that among opioid users the rate of progression to dependence was quicker among women than men [40], and women experience more craving for opioids. Psychological and or emotional distress among women have been found to be significant risk factors for excessive use of prescription opioid use [41].

Findings from the multivariable model suggest that age was a clear predictor of doses dispensed per person with a clear trend in increased doses with increasing age, peaking for those in the 65+ year age-group. This is likely due to the high prevalence of pain among older people. Interestingly, significantly more quantities (in DDD) were used in LGAs that are categorized as “rural” than in LGAs categorized as “urban”. This model also indicates that doses dispensed per person increased with increasing disadvantage. These observations suggest that social determinants which impact on a range of aspects of the health of Australians also affect levels of pharmaceutical drug use [42]. People living in disadvantaged areas are likely to have relatively poor health and well-being. Being in disadvantaged areas also means that they are more likely to have concession cards and enjoy lower copayments under the Australian subsidized medicine program and so may be more likely to access more medications than others [43].

Geographical variations in dispensing were notable – ranging from 5.01 to 45.58 DDDs (in 2015 adjusted for age and sex) – with most of the areas with very high dispensing outside of the major metropolitan cities. This could reflect a lack of alternative treatment/management options for pain in rural and remote locations. It is also possible that prescribing practices among doctors are relatively relaxed in remote areas and possibly reflects the long-term doctor-patient relationship and a socio-cultural approach to healthcare provision in rural areas [44]. Higher levels of dispensing, however, do not necessarily indicate an inappropriate or problematic utilization. Instead, these findings warrant further and area-based research, including qualitative research, to further explore factors leading to higher dispensing in these areas. It is also important to remember that an area being identified as having certain characteristics does not imply all the individuals living in the area possess these characteristics and could represent what has been termed an “ecological fallacy” [45].

This study did not aim to measure the consequences of the growing use of prescription opioids. However, it should be noted that excessive use of prescription opioids and associated harms are a major public health problem in many countries. Dependence and other side effects of long term opioid use and death from overdose have received substantial coverage recently in electronic and print media, prompting policy interventions. Prescription Shopping Information Service is one such intervention, which allows prescribers and suppliers to identify potential prescription shoppers and look at ways to reduce patient need for such shopping [46]. Despite these concerns prescription opioid use has continued to increase in Australia.

Nationwide implementation of a real-time prescription drug-monitoring program (PDMP) and access to the database from both doctors and pharmacists at point-of-care is another measure that may reduce excessive and undue use of opioids [47–50]. Current initiatives of PDMP are taken by the individual states/territories. Tasmania was the first jurisdiction in Australia to implement a program of PDMP. Recently the Victorian state government funded a real-time PDMP [51]. The Australian Government is reported to be interested in rolling out PDMP, and is waiting for each state and territory to commence using it [52]. A nationwide initiative of PDMP is likely to be more effective and sustainable, as interoperability of these state-based systems using different software may be problematic [53]. This will also help prevent the cross-border fraudulent access to opioids. However, the evaluation of PDMP is crucial to ensure therapeutic and social aims of the program can be met and to avoid any unintended consequences such as patients shifting to illicit drugs or other prescription drugs (e.g., benzodiazepines) and overly cautious prescription of drugs when they are clinically indicated.

### Limitations

Our study has a number of limitations. Firstly, complete data for only 3 years were available for assessing the temporal trend. Also, opioids dispensed through State Government hospitals or those not listed in the PBS/RPBS schedule were not captured in this dataset. However, overall it should be a small quantity, and is unlikely to have significant impact on our major findings. The DDD metric does not necessarily correspond to the recommended or prescribed daily dose.

## Conclusion

Spatial and temporal variations in prescription opioid dispensing were identified for NSW during the study period (1 January 2013–31 December 2015). Very high and high levels of dispensing in terms of DDDs were recorded in a number of LGAs, with most with relatively high dispensing located outside the major cities. Age, sex and neighborhood level disadvantages are three significant factors in doses dispensed. There are likely to be other potential factors which need to be examined further. This study provides a basis for such further investigation. Given that long-term use of opioid has substantial side effects including dependence and given that over-use of opioid is a major public health problem, it is important that research facilitate understanding of behavior in small areas to enable targeted interventions. A real time prescription drug monitoring program may also play a role in reducing problematic use of prescription opioids, however, such programs must be designed well and evaluated rigorously to ensure therapeutic aims are met and unintended consequences are avoided.

## Abbreviations

DDD: Daily Defined Dose
LGA: Local Government Area
NSW: New South Wales
PBS: Pharmaceutical Benefit Scheme
PDMP: Prescription Drug Monitoring Programme
RPBS: Repatriation Pharmaceutical Benefits Scheme
SEIFA: Socio-Economic Indexes for Areas
